# Intact Type I Interferon Production and IRF7 Function in Sooty Mangabeys

**DOI:** 10.1371/journal.ppat.1003597

**Published:** 2013-08-29

**Authors:** Steven E. Bosinger, Zachary P. Johnson, Kathryn A. Folkner, Nirav Patel, Tayebeh Hashempour, Simon P. Jochems, Perla M. del Rio Estrada, Mirko Paiardini, Rongtuan Lin, Thomas H. Vanderford, John Hiscott, Guido Silvestri

**Affiliations:** 1 Divison of Microbiology and Immunology, Emory Vaccine Center, Yerkes National Primate Research Center, Atlanta, Georgia, United States of America; 2 Non-Human Primate Genomics Core, Yerkes National Primate Research Center, Robert W. Woodruff Health Sciences Center, Emory University, Atlanta, Georgia, United States of America; 3 Division of Cognitive and Developmental Biology, Yerkes National Primate Research Center, Atlanta, Georgia, United States of America; 4 Lady Davis Institute-Jewish General Hospital, McGill University, Montreal, Quebec, Canada; 5 Vaccine and Gene Therapy Institute of Florida Port Saint Lucie, Florida, United States of America; National Institute of Allergy and Infectious Diseases, National Institutes of Health, United States of America

## Abstract

In contrast to pathogenic HIV/SIV infections of humans and rhesus macaques (RMs), natural SIV infection of sooty mangabeys (SMs) is typically non-pathogenic despite high viremia. Several studies suggested that low immune activation and relative resistance of CD4+ central memory T-cells from virus infection are mechanisms that protect SMs from AIDS. In 2008 it was reported that plasmacytoid dendritic cells (pDCs) of SMs exhibit attenuated interferon-alpha (IFN-α) responses to TLR7/9 ligands *in vitro*, and that species-specific amino acid substitutions in SM Interferon Regulatory Factor-7 (IRF7) are responsible for this observation. Based on these findings, these authors proposed that “muted” IFN-α responses are responsible for the benign nature of SIV infection in SMs. However, other studies indicated that acutely SIV-infected SMs show robust IFN-α responses and marked upregulation of Interferon Stimulated Genes (ISGs). To investigate this apparent disparity, we first examined the role of the reported IRF7 amino acid substitutions in SMs. To this end, we sequenced all IRF7 exons in 16 breeders, and exons displaying variability (exons 2,3,5,6,7,8) in the remainder of the colony (177 animals). We found that the reported Ser-Gly substitution at position 191 was a sequencing error, and that several of the remaining substitutions represent only minor alleles. In addition, functional assays using recombinant SM IRF7 showed no defect in its ability to translocate in the nucleus and drive transcription from an IFN-α promoter. Furthermore, *in vitro* stimulation of SM peripheral blood mononuclear cells with either the TLR7 agonist CL097 or SIV_mac239_ induced an 500–800-fold induction of IFN-α and IFN-β mRNA, and levels of IFN-α production by pDCs similar to those of RMs or humans. These data establish that IFN-α and IRF7 signaling in SMs are largely intact, with differences with RMs that are minor and unlikely to play any role in the AIDS resistance of SIV-infected SMs.

## Author Summary

Sooty mangabey (SM) monkeys are an important model for studying HIV disease processes because they do not develop AIDS when infected with SIV, a primate version of HIV. The reasons why SIV-infected SMs remain healthy are not completely understood, but are related to reduced activation of the immune system, and to their ability to shut off a strong antiviral response (Interferon-alpha), that is present during early infection but disappears over time. The interferon-alpha response is an essential component of the host immune response to viral infections, but may contribute to AIDS progression if it persists indefinitely, as occurs in HIV-infected humans and AIDS-susceptible macaque monkeys. In this study, we found that, contrary to a previous report, SMs had an intact interferon response, and SM plasmacytoid dendritic cells (pDCs), a cell-type specialized in producing interferon, were not deficient in their responses to SIV. We also show that IRF7, a molecule essential to initiate the interferon-alpha response, maintains function in SMs compared to humans and macaques. These data provide novel information on how SIV-infected SMs avoid AIDS despite high levels of virus, and support the hypothesis that immuno-regulatory mechanisms underlie their ability to rapidly shut-off the interferon-alpha that occurs during early infection.

## Introduction

In contrast to human immunodeficiency virus (HIV) infection of humans and experimental simian immunodeficiency virus (SIV) infection of Asian macaques, SIV infection of African monkeys that are natural hosts, such as sooty mangabeys (SM), is typically non-pathogenic despite high virus replication [1], [2]. Understanding the mechanisms underlying how SMs are able to avoid AIDS remains an area of active investigation [3].

Type I interferons, including IFN-α, are a family of cytokines that play a central role in the innate antiviral response mediated by different cell types, and in particular plasmacytoid dendritic cells, pDCs [4]. The production of type I IFNs is induced by numerous innate signaling pathways (including TLRs, NLR, RLRs, etc) and results in the expression of hundreds of antiviral effector genes that are collectively referred to as Interferon Stimulated Genes (ISGs) [5]–[7]. Pathogenic HIV infection of humans and SIV infection of macaques are associated with a strong type I interferon response that persists during the chronic phase of infection [8]–[11]. While exogenous IFN-α administration exerts a clear antiviral effect in vivo in both pathogenic and non-pathogenic infections [12]–[14], an elevated type I IFN response has also been proposed as an immunopathogenic mechanism [15]–[17] and reported as a marker of poor immunologic response to antiretroviral therapy [18]. As such, the pathophysiological consequences of type I interferon responses during pathogenic HIV/SIV infections are complex and not fully understood.

An article published in Nature Medicine in 2008 by Mandl et al. reported that pDCs of SMs exhibit a muted *in vitro* production of type I interferon (IFN) in response to Toll-like receptor (TLR)-7/9 ligands and SIV, and attributed this observation to amino acid substitutions specific to SMs in the transactivation domain of the Interferon regulatory factor (IRF) 7 signaling molecule [19]. Based on these findings, the authors proposed that this muted type I IFN response to SIV is the key mechanism protecting SIV-infected SMs from developing chronic immune activation in response to the virus, and ultimately from development of AIDS [19]. If confirmed, these data would be of significant scientific impact as they would indicate that the evolutionary pressure posed by SIV in the SM innate immune system has resulted in the functional amputation of a signaling axis (i.e., the IRF7/type I IFN) that is evolutionarily conserved and known to play a major role in containing the replication of RNA viruses in many models of infection [20]. On the other hand, several independent studies from other groups suggested that acute SIV infection of SMs is in fact associated with a widespread innate and adaptive immune response to the virus with massive upregulation of dozens of interferon-stimulated genes (ISGs) [10]; production of IFN-α and IFN-β in the lymph nodes of acutely infected SMs [21]; and detection of plasma IFN-α and IP10/CXCL10 during acute SIV infection [22]. Of note, this immune response and ISG upregulation is controlled within a few weeks of the initial infection in SIV-infected SMs, while chronic immune activation and persistent type I IFN responses are present during pathogenic HIV/SIV infections of humans and rhesus macaques [8]–[10], [17].

In this study, we sought to investigate the apparent discrepancy between the largely *in vitro* data reported in Mandl et al, and the *in vivo* data indicating a strong type I IFN response during acute SIV infection of SMs. To this end, we performed a series of experiments investigating the overall functionality, in SM-derived PBMCs and pDCs, of the innate immune pathway involving TLR7/9 stimulation, IRF7 activation, and type I IFN gene expression and protein production. We found that: (i) most of the SM-specific amino acid substitutions in IRF7 originally reported by Mandl et al., were either minor alleles or an artifact of sequencing error; (ii) IRF7 of SMs is fully functional and retains the ability to translocate in the nucleus and transactivate IFNA genes; (iii) SM PBMCs upregulate type I IFN mRNA expression by 500–800 fold in response to SIV_mac239_ or TLR7 ligand CL097; and (iv) SM pDCs show a robust production of IFN-α in response to CL097 or SIV_mac239_ by intracellular cytokine staining. Based on these results we concluded that IFN-α and IRF7 signaling in SMs are largely intact, and that any differences with RMs are minor and highly unlikely to play any role in the AIDS resistance of SIV-infected SMs.

## Results

### IRF7 sequence in Sooty Mangabeys

The SM IRF7 gene sequence published in Mandl et al. [19] was the first description of this important innate immune gene in a natural SIV host species. Of note, that sequence refers to data derived from a single animal (FHy) using cDNA as a template. SM-IRF7 contains 10 exons. To accurately assess the species-wide variation of the IRF7 gene in SMs, we initially sequenced all 10 exons in 16 breeder animals representative of the genetic variability in the colony housed at the Yerkes Primate National Research Center (YNPRC) based on pedigree. Sequencing was performed on genomic DNA in both directions, and any overlapping loci displaying ambiguity were re-sequenced. Six (exons 2, 3, 5, 6, 7, and 8) displayed variation at the amino acid level within 16 breeder animals initially sequenced. No variation was observed in four exons (1,2,9 and 10) in the breeders. To establish the colony-wide distribution of polymorphisms within variant-containing exons, we then sequenced the six exons displaying variability in the remainder of the colony (177 animals). We were able to generate sequence data in all 177 animals for exons 2, 3, 5, 7 and 8, but were only able to sequence exon 6 in 50 animals. No further sequencing efforts were undertaken for exons 1, 4, 9 or 10 as no variation within these regions was observed in Mandl et al [19] or within our initial sequencing of breeders. A summary of the number of animals sequenced for each exon is detailed in **Supplementary Table S1**. Importantly, we also re-sequenced SM-IRF7 in the animal (FHy) from which the sequence reported in Mandl et al. [19] was derived.

Our sequence analysis indicated that, out of the seven SM-specific amino acid substitutions reported in Mandl et al. [19] one substitution (Ser-to-Gly in position 191) is a sequencing error and four others are not fixed in the SM population, with two being present in homozygosity only in a minority of animals (**Table 1**). It should be noted that of the seven SM-specific amino acid substitutions originally reported, the Ser-to-Gly at position 191 was the most potentially disruptive, since the serine in this position is highly conserved across species, being present in humans, macaques, mice, rats and various non-human primates. Upon re-sequencing the SM colony, we did not observe this purported genotype in any of the 16 breeder animals, nor in the 177 non-breeders. We also re-sequenced animal FHy, from which the SM-IRF7 sequence was derived in the original study by Mandl, and again, did not observe the S191G substitution. These data indicate that serine is present at position 191 in the entire SM colony. While this amino-acid substitution would have represented a good candidate for phenotypic differences in SMs, our results indicate that the observation of this polymorphism in Mandl et al. [19] is an artifact of a sequencing error.

**Table 1:** Genotype frequencies of predicted amino acid substitutions in Sooty Mangabey IRF7.

Amino Acid position^a^	165	191	203	252	256	268	413
**Human IRF7**	A	S	G	A	T	A	Q/R
**Rhesus IRF7**	A	S	G	T	T	A	Q
**Mangabey IRF7 - Mandl ** ***et al.***	G	G	A	V	A	V	R
**Mangabey IRF7 - Mandl ** ***et al.*** ** resequencing**	G	S	A	V/A	A	V	R
**Sooty Mangabey Population Genotype Frequency (%)**	G/G (78%)	S/S (100%)	A/A (100%)	V/A (43%)	A/T (30%)	V/A (25%)	R/R (100%)
	S/G (22%)			A/A (33%)	A/A (28%)	V/V (73%)	
				V/V (24%)	T/T (42%)	A/A (2%)	

a aa position in SM IRF7.

Colony-wide re-sequencing also demonstrated that other non-synonymous nucleotide changes reported by Mandl et al. [19] either represented minor variants within the colony or resulted in conservative amino acid changes. At position 252, the SM sequence was originally reported to be valine, divergent from alanine in humans and threonine in rhesus macaques (**Table 1**). Upon resequencing animal FHy, this position was found to be heterozygous (alanine/valine). Sequencing 50 SMs for exon 6 showed that 24% were V/V homozygous, 33% A/A homozygous, and 43% A/V heterozygous. Given the variability at this locus among SMs, this substitution is unlikely to have major effects on immune activation, as all published data indicate that the low immune activation of SIV-infected SMs is a general phenomenon among these animals. Position 256 was reported to be a Thr-to-Ala substitution in SMs. Resequencing showed that animal FHy is indeed A/A homozygous in this position. However, our sequencing of 50 animals and found that only 28% were A/A, 30% heterozygous A/T, and 42% homozygous T/T (and therefore identical to humans and macaques). Position 165 was reported as an Ala-to-Gly substitution in SMs; however, G/G homozygosity was found in 78% of SMs, with 22% G/A heterozygosity. While the reported sequence of FHy for this position is correct, our data indicate that a significant polymorphism is present at the population level in SMs. At position 268, the SMs were reported to have an Ala-to-Val substitution. Resequencing demonstrated that 73% of the animals were homozygous for V/V, 25% V/A heterozygous, and 2% homozygous A/A. In summary, our work demonstrates that, of the seven purportedly SM-specific IRF7 amino-acid substitutions described in Mandl et al. [19], only two are actually fixed in the SM population (i.e., Ala-to-Gly at position 203, which is highly conservative, and Gln-to-Arg at position 413). While R at position 413 was fixed in the SM colony, the locus is not fixed in humans, in whom R is present with 28% minor allelic frequency [23].

As mentioned above, low immune activation is common to virtually all SIV-infected SMs. However, variations among individual animals have been described [24], [25]. To determine whether the observed single nucleotide polymorphisms in the IRF7 sequence impact on the phenotype of SIV infection in SMs, we tested for the presence of statistically significant relationships between the observed SNPs and markers of SIV disease progression. We found that none of the observed IRF7 polymorphisms in SIV-infected SMs showed a significant association with viral load, CD4+ T cell counts, or immune activation (**Table 2**).

**Table 2:** Association of SM polymorphisms with markers of SIV disease progression.

	AA 165^a^	AA 252	AA 256	AA 268
**Plasma Viral Load (copies/ml)**	*G/G* 1.7×10^5^±2.9×10^4^	*V/V* 3.9×10^5^±1.8×10^5^	*A/A* 1.7×10^5^±8.4×10^4^	*V/V* 1.4×10^5^±2.8×10^4^
	*S/G* 1.4×10^5^±4.2×10^4^	*A/A* 1.0×10^5^±3.3×10^4^	*T/T* 1.4×10^5^±3.4×10^4^	*A/V* 2.2×10^5^±4.8×10^4^
	(1,64) p = 0.59	*V/A* 1.6×10^5^±5.1×10^4^	(1,15) p = 0.69	(1,56) p = 0.19
		(2,13) p = 0.04^b^		
**Peripheral CD4+ count (cells/ul)**	*G/G* 1025±76	*V/V* 1000±296	*A/A* 924±236.9	*V/V* 1163±98
	*S/G* 1219±103	*A/A* 1508±136	*T/T* 1240±144	*A/V* 972±82
	(1,66) p = 0.16	*V/A* 1002±173	*A/T* 881±395	(1,59) p = 0.16
		(2,15) p = 0.07	(2,16) p = 0.41	
**CD4+Ki67+ (%)**	*G/G* 3.0±0.2	*V/V* 2.9±0.8	*A/A* 2.9±0.3	*V/V* 3.3±0.23
	*S/G* 3.5±0.2	*A/A* 3.1±0.2	*T/T* 3.1±0.2	*A/V* 3.12±0.26
	(1,66) p = 0.22	*V/A* 2.9±0.32	*A/T* 1.9±0.4	(1,59) p = 0.57
		(2,15) p = 0.85	(2,16) p = 0.10	
**CD8+ Ki67+ (%)**	*G/G* 3.2±0.3	*V/V* 2.5±1.5	*A/A* 3.0±0.6	*V/V* 3.3±2.7
	*S/G* 3.7±0.3	*A/A* 2.4±0.3	*T/T* 2.6±0.3	*A/V* 3.4±2.5
	(1,64) p = 0.36	*V/A* 3.0±0.3	*A/T* 2.5±0.5	(1,57) p = 0.8
		(2,14) p = 0.5	(2,16) p = 0.79	
**CD123+ pDCs (%)**	*G/G* 11.0±0.8	*V/V* 6.0±1.0	*A/A* 11.3±2.2	*V/V* 11.9±1.1
	*S/G* 13.7±2.3	*A/A* 12.9±2.62	*T/T* 12.5±3.4	*A/V* 11.1±1.7
	(1,64) p = 0.16	*V/A* 16.3±4.9	*A/T* 19.0±0.0	(1,57) p = 0.70
		(2,14) p = 0.45	(2,16) p = 0.61	

a mean and S.E. are indicated for denoted genotypes; degress of freedom are indicated by parantheses.

b significance threshold corrected for multiple hypothesis testing (Bonferroni) is 0.01.

### Sooty Mangabey IRF7 retains IFNA transactivation and nuclear translocation activity

**Figure 1:**
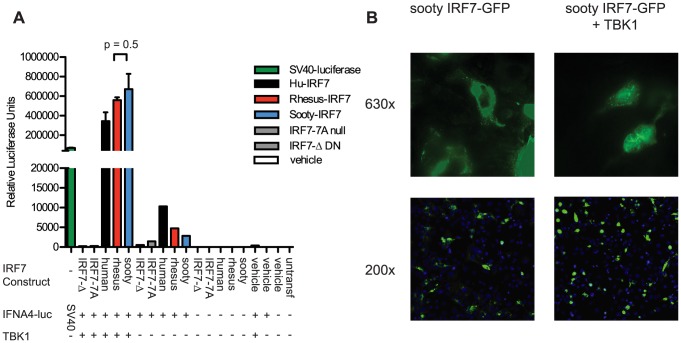
Transactivation of IFNA4 promoter and nuclear localization by Sooty Mangabey IRF7. (A) HEK293 cells were transfected with the luciferase reporter plasmid containing the human IFNA4 promoter, TBK1 and IRF7 constructs from human, rhesus, and sooty mangabeys, or vehicle, as indicated. Luciferase expressed from the SV40 promoter was transfected as a positive control. Luciferase activity was measured 24 h after transfection. Values represent the average of triplicate wells for Rhesus and Sooty-IRF7 and duplicate wells for the remaining samples. Data are representative of three individual experiments. (B) COS-7 cells were transfected with Sooty-IRF7-GFP and TBK1, or with vehicle for 24 hrs, then stained with DAPI. Magnification is indicated to the right of panels. Data are representative of three experiments.

While our extensive sequencing of SM-IRF7 did not reveal any difference with either human or rhesus macaque IRF7 genes that would predict a significant loss of function, these sequencing data do not provide per se any functional information about SM-IRF7. For this reason, we elected to next assess the transactivation potential of SM-IRF7 on an IFNA promoter. We synthesized and cloned into the expression vector pCMV2 a full-length SM-IRF7 gene sequence identical to that of animal FFz, which contained the most representative alleles found in the SMs housed at the Yerkes colony. The ability of SM-IRF7 to initiate IFNA transcription was compared to that of RM-IRF7, human IRF7, and two loss-of-function mutants, using a well-described reporter system in which IRF7, a hu-IFNA4 reporter construct and the activating kinase TBK1 are co-expressed in HEK 293 cells [26]. As shown in Figure 1A, co-expression of human, RM, and SM-IRF7 with the IFNA4 reporter construct alone yielded moderate induction of luciferase activity, consistent with the basal activity that has been reported previously for hu-IRF7 [27]. However, co-transfection of SM-IRF7 together with the IFNA4 reporter and the activating kinase TBK1 resulted in strong induction of transcriptional activity that was >600,000-fold over background (**Figure 1A**). Importantly, we did not observe any significant difference between the transactivation activity of SM-IRF7 and RM-IRF7. The observed activity of SM-IRF7 was slightly higher than that of human IRF7 (**Figure 1A**). In addition, we compared SM-IRF7 activity against two loss-of-function IRF7 mutants, IRF7-7A, which contains Ser-to-Ala substitutions in the serine-rich domain, and IRF7-Δ, a dominant-negative mutant missing AA 7-101 of the DNA-binding domain. If the few actual species-specific substitutions in SM-IRF7 impaired its ability to initiate transcription from the IFNA promoter, we would predict that its activity would be closer to the range of the loss-of-function mutants. However, SM-IRF7 transactivation activity was >1000-fold higher than the mutants, and was in general equivalent to, or greater than, human or RM IRF7 constructs (**Figure 1A**).

**Figure 2:**
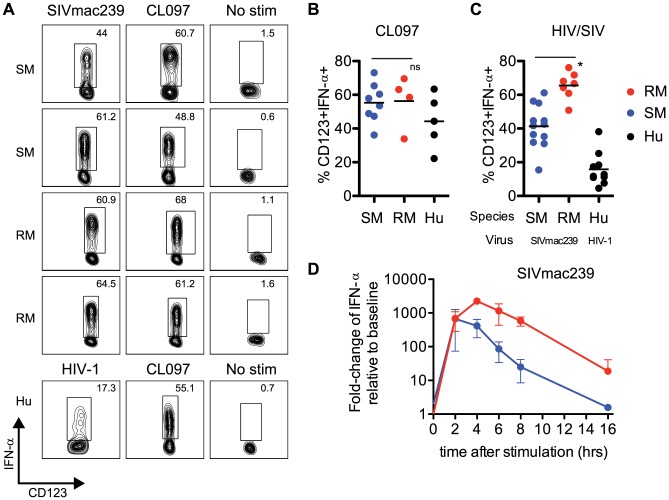
Sooty mangabey pDCs produce IFN-α in response to TLR7 agonists and SIVmac239. (A) PBMCs from SIV-negative SMs and SIV-negative RMs were incubated for 18 hr with 10 µM CL097 or 3 µg/ml SIVmac239 and stained for intracellular IFN-α. The lower panels depict uninfected human PBMCs stimulated with 10 µM CL097 or 3 µg/ml AT2 HIV-1. Insets denote percentages of IFN-α+ pDCs within the pDC population. (B) Cumulative data for CL097 stimulations, average percentage of pDCs expressing IFN-α are denoted by horizontal bars. (C) Percentage of IFN-α+ pDCs after 18 hr stimulation with AT2 SIV_mac239_, or AT2 HIV-1 (for human PBMCs). Means are shown by horizontal bars. The species (RM, SM or HU) from which PBMCs were prepared and virus strain used for stimulation is depicted below the X-axis. (D) RNA production of IFN-α in PBMCs from SMs and RMs was assessed using qPCR. Fold-changes were calculated as relative to unstimulated cells, after GAPDH normalization. Experiments are the average of three animals, each measured in triplicate wells. All viral stimulation experiments (ICS and qPCR) were performed using AT2-inactivated preparations of SIV_mac239_ or HIV-1.

Previous studies have demonstrated that viral infections can induce the nuclear translocation of IRF7 in transfected cells [28], [29] and in purified pDCs [30]. Studies of IRF7 nuclear retention activity have shown that mutants containing deletions of AA247-415, AA247-305, AA417-440, or S477A/S479A substitution mutations can abrogate nuclear translocation. As some of the species-specific amino acid substitutions in SM-IRF7 are located in these regions, we considered that the possibility that they may abrogate the ability of SM-IRF7 to enter the nucleus after activation. We thus tested this hypothesis using an assay that employs co-transfection of IRF7-GFP fusion protein with TBK1 [26]. Transfection of SM-IRF7-GFP alone resulted in cells displaying a predominantly cytoplasmic localization, with the transfected molecule being excluded from DAPI-stained nuclei (**Figure 2B**). However, 24 hrs after co-transfection with TBK1, the majority of cells expressing of SM-IRF7-GFP were clearly localized in the nucleus (**Figure 2B**). Collectively, these data indicate that the observed species-specific substitutions in SM-IRF7 do not confer a defect in its ability to transactivate IFNA expression relative to humans or RM IRF7, nor do they abrogate the ability of SM-IRF7 to translocate to the nucleus after activation.

### pDCs from Sooty Mangabeys respond to SIV_mac239_ with robust levels of IFN-α production

Our sequence and functional data indicated very clearly that IRF7 signaling in SMs was intact, and comparable to that of humans or RMs. To further investigate potential intrinsic defects in the type I IFN response in SMs, we examined the production of IFN-α by pDCs from uninfected SMs (defined as Lineage-neg, HLA-DR+, CD123+ cells, gating strategy shown in **Supplementary Figure S1**) after ex vivo stimulation with aldrithiol-2 inactivated (AT-2) SIV strains (i.e., SIV_mac239_ and SIV_smmE543.1_) or TLR7 ligands. We found that after overnight culture with SIV_mac239_, production of IFN-α by SM pDCs was quite robust, with an average frequency of IFN-α-producing pDCs of 41.3% (**Figure 2A,C**). The lowest responder SM that we observed had a frequency of 15.4% of IFN-α-producing pDCs in response to SIV_mac239_, and the highest responder showed a frequency of 61.2% (**Figure 2A**). Of note, SM pDCs incubated without virus or with microvesicles always produced <2% background production of IFN-α. When SM PBMCs were incubated with the SM-derived strain AT-2 inactivated SIV_smmE543.1_, we also observed robust IFN-α production in most animals, with the highest responder having a frequency of 42.7% pDCs staining positive for intracellular IFN-α. Average production of IFN-α in SM pDCs in response to SIV_smmE543.1_ was 21.9%, with two animals showing low responses at ∼5% (**Supplementary Figure S2**). In the next set of experiments, we incubated PBMCs from SMs with CL097, a derivative of the synthetic TLR7 agonist R848, and observed a similarly robust induction of IFN-α by pDCs (**Figure 2A,B**). Indeed, the average level of IFN-α induction in pDCs following CL097 stimulation was indistinguishable between SMs compared to RMs (55.0% and 56.2% of IFN-α-producing pDCs, respectively). While IFN-α production in response to SIV_mac239_ was higher in RMs than in SMs (65.5% vs 41.3% of IFN-α-producing pDCs p<0.05), the type of response observed in SMs is far from “muted” and in fact is actually higher than what we observed in human pDCs incubated with AT-2 inactivated HIV (**Figure 2C**). Similarly, the IFN-α response in pDCs to SIV_smmE543.1_ was higher in RMs than SMs (41% vs 21%), however the response in SMs was overall quite robust. To compare the kinetics of IFN-α expression in SMs and RMs, we next conducted a series of time course experiments and measured the level of IFN-α mRNA in PBMCs at various time points after stimulation with AT-2 SIV_mac239_. As shown in **Figure 2D**, we observed that mRNA induction was very high in both species two hours after incubation and virtually equivalent in magnitude (average fold-change relative to unstimulated: SM 669-fold, RM 682-fold). However, after two hours of stimulation, the kinetics diverged between the two species, with RMs peaking at four hours post-stimulation whereas SMs started to decline. After overnight stimulation, IFN-α levels returned to baseline in the SM (1.6-fold) but remained elevated in the RMs (18-fold). Interestingly, these data are consistent with the strong pattern of IFN-α mRNA induction in SMs described in Mandl et al [19]. It is tempting to speculate that the observation of a similarly strong but more transient IFN-α upregulation in SMs as compared to RMs may play a role in the rapid down-modulation of the type I IFN response that occurs *in vivo* during acute SIV infection of SMs.

### Sooty Mangabeys produce IFN-β in response to SIV and TLR7 ligands

**Figure 3:**
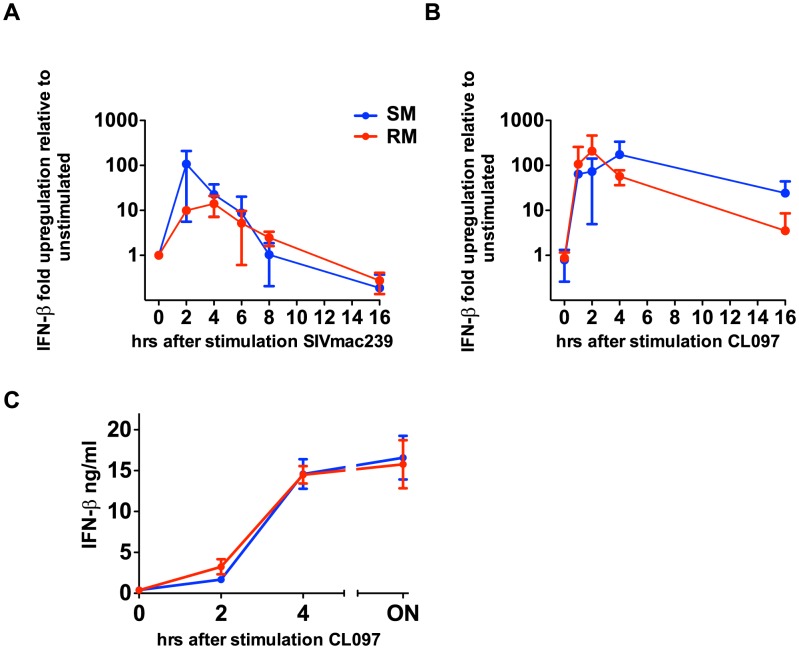
SIVmac239 and TLR7 agonists induce IFN-β production by SMs. PBMCs were stimulated with CL097 (A) or SIV_mac239_ (B), RNA was harvested at indicated time points and quantitated by qPCR. Fold-changes were normalized by GAPDH, and are expressed as relative to unstimulated replicates. Values are averages of three animals; values for individual animals were averaged from triplicate wells. (C) PBMCs from RMs and SMs were incubated for CL097 and supernatants were harvested at the times indicated and IFN-β was assessed by ELISA.

During the acute phase of SIV infection, SMs produce high levels of type I IFNs and show massive upregulation of ISGs, which can be induced by any type I IFN (α,β,ω,λ) [10], [21]. To test the possibility that SMs can produce other non-α type I IFNs in response to TLR7 stimulation, we measured the level of IFN-β gene expression at the RNA level in PBMCs derived from both SMs and RMs after *in vitro* stimulation with the TLR7 ligand CL097 as well as SIV_mac239_. It should be noted that, in these experiments, we could not link IFN-β production to pDCs due to lack of an IFN-β-specific monoclonal antibody that can be used for flow cytometric analysis. As shown in **Figure 3**, both the TLR7 ligand CL097 and SIV_mac239_ induced a marked upregulation of IFN-β gene expression. Concurrently, we also observed robust upregulation of IFN-β protein in the supernatants of PBMCs stimulated with CL097. While multiple cell types may be contributing to IFN-β production, these data are consistent with previously published results showing high levels of IFN-β production by pDCs in the lymph nodes of SIV-infected SMs during the acute phase of infection [21], and indicate that, in SMs, the ability to produce additional type I IFNs after TLR7 stimulation is largely intact.

## Discussion

In stark contrast to pathogenic HIV and SIV infections of humans and macaques, respectively, SIV infections of natural host species such as the sooty mangabeys (SM) are typically non-pathogenic despite high viremia. The mechanisms by which SIV-infected SMs avoid AIDS are complex and only partly understood, but appear to be related to the absence of chronic immune activation and the relative preservation of CD4+ central-memory T cells from direct virus infection [31]. Of note, the attenuated immune activation of chronically SIV-infected SMs occurs as a result of a rapid down-modulation of a vigorous innate and adaptive immune response that is observed during the first weeks of infection [10], [32], [33].

An influential study published in 2008 by Mandl et al [19] reported that SMs exhibit a near-complete deficiency in the ability of pDCs to produce IFN-α in response to SIV or TLR7 ligands *in vitro*. In this study, the defective IFN-α production was attributed to species-specific differences at the amino acid level of the IRF7 signaling molecule between SMs and “pathogenic” hosts such as humans and rhesus macaques [19]. The authors concluded that a “muted” type I IFN response to SIV by pDCs is the main reason why SIV-infected SMs avoid chronic immune activation and do not progress to AIDS. This study drew considerable interest, as it would have represented, if confirmed, the first example of a species-specific adaptation to a chronic virus infection that occurs through a genetically determined abrogation of a key innate immune pathway, i.e., the TLR7/9-IRF7-type I IFN pathway. However, subsequent studies of the *in vivo* immune response to SIV during acute infection of SMs showed a vigorous, albeit transient, type I IFN response in the blood and lymph nodes of these animals [10], [21]. In the current study, we have found that several of the published experimental results could not be replicated, and have provided extensive evidence that, in fact, SMs exhibit an intact IRF7 sequence and function, and most importantly, are capable of strong type I IFN production in response to SIV and TLR-7 ligands.

We first set out to establish the genetic distribution of the reported IRF7 polymorphism within the SM population housed at the Yerkes Center (total of 177 animals), and found that the most potentially disruptive mutation, S191G, reported by Mandl et al [19] was a sequencing error, and other reported species-specific amino acid substitutions in SM IRF7 are allelic variants whose presence or absence has no impact whatsoever on the phenotype of SIV infection in terms of virus replication, CD4+ T cell counts, level of immune activation or disease progression. The inaccurate and/or incomplete information on the gene sequence of SM IRF7 presented in Mandl et al [19] reflects the fact that only one animal was sequenced, and that the sequence was generated from cDNA derived from mRNA rather than direct sequencing of genomic DNA. The reasons why such a strong conclusion was reached based in an extremely limited set of data are unclear. In any event, the current set of data reflects a truly comprehensive analysis of the SM-IRF7 gene sequence and allelic polymorphisms that should from now on represent the reference dataset for those interested in this field of investigation.

In this study, we were able to confirm that two of the reported substitutions located in a region of IRF7 that, based on human studies, may impact on pDC function [28] were indeed fixed in the SM colony (G203A and Q413R). While the G203A allele is highly conservative and likely to be of limited impact, the Q413R substitution is more interesting. Of note, the R allele is expressed at an estimated 28% prevalence in the human population [23], although its presence was not linked to diminished type I IFN production by pDCs [34]. The Q413 allele was also examined in the context of systemic lupus erythematosus (SLE), in which one report suggested an association between this allele and an increased susceptibility to SLE [35], and subsequent work did not replicate these findings [36]. Given these findings, we felt that it was important to directly test the ability of SM-IRF7 to transactivate an IFNA promoter. We found that the consensus SM-IRF7 is perfectly capable of efficiently initiating IFNA transcription and that the level of transactivation is equivalent or higher to that of humans and RMs. Of note, our assay system utilized TBK1 to activate the transfected IRF7 constructs. IRF7 is primarily activated through two pathways: endosomal TLR7/9 and the ‘intrinsic’ or RIG-I related pathway [5]. pDCs exclusively use the TLR7/9 pathway to sense RNA viruses [37], in which IRF7 is phosphorylated by IKKα in association with TRAF6, MYD88 and other accessory molecules [38], [39]. Non-pDC cell types predominantly use the RIG-I pathway, which activates IRF7 via TBK1 [37], [40]. Our choice of *in vitro* system was based on comparative robustness; although IKKα is more directly relevant to pDCs, it requires co-transfection of additional adaptor proteins and typically results in a modest signal [27], [38]. The TBK1 reporter system we chose does not require multiple accessory proteins system [26], [38] and has a stronger signal than other adaptor proteins such as MYD88 or TRAF6 [39]. Nevertheless, these data indicate that SM-IRF7 maintains its primary function, namely induction of transcriptional activity.

During a viral infection, rapid induction of innate immunity is necessary to limit viral burden and spread, until an effective adaptive response can be mounted. Equally important is the ability to ‘rein in’ the response, as unabated activation of the TLR and IFN systems is deleterious to the host, and may lead to immunopathology [41]. While our data has demonstrated that SM-IRF7 maintains the ability to efficiently transactivate IFNA promoter activity, it is possible that the SM-specific amino acids that are divergent from humans and RMs could influence other aspects of IRF7 activity, in particular its negative-regulation. Although no studies to date have examined the role of human genetic polymorphisms in the regulation of IRF7 activity, recent work has uncovered molecular strategies by which viruses inhibit IRF7 activity and, conversely, by which mammalian cells self-regulate the IFN response by modulating IRF7 at the transcriptional and post-transcription levels [42]. Litvak et al demonstrated that the transcription factor FOXO3 acts to suppress IRF7 transcription by enhancing deacetylation of the IRF7 promoter [43]; and Lee and colleagues have demonstrated that regions in the 5′ UTR of IRF7 mRNA are targeted for degradation by the ISG OASL in a negative feedback cycle [44]. In previous work, we showed that *in vivo* SIV infection of SMs is characterized by an initial, widespread IFN response that is rapidly down-regulated within a few weeks from the initial infection [10]. In this study, we observed *in vitro* that the production of IFNA mRNA in response to SIV also declines more rapidly in SMs than RMs, suggesting that the resolution of the ISG response observed during SIV infection may be due to a more rigid control of IRF7 activity. While this study focused on the coding regions of SM-IRF7, it will be interesting to evaluate if interspecies differences in the 5′ UTR of IRF7 alter the regulatory feedback loops controlling its activity.

Having established that the IFNA transactivation activity of SM-IRF7 is intact, we next investigated the ability of SMs to produce type I IFNs in response to SIV, HIV or the TLR7 ligand CL097. We found that SM pDCs were able to robustly produce high levels of IFN-α in response to CL097, SIV_mac239_ and SIV_smE543.1._ We observed that SM pDCs produced similar levels of IFN-α compared to RMs when cultured with CL097, and were only slightly reduced for SIV_mac239_. These data are in direct contrast with the results of Mandl et al, who found an average of ∼5% pDCs producing IFN-α in response to SIV_mac239_ [19]. One possible explanation for this discrepancy is that the addition of BFA after 8 hours of stimulation has resulted, in Mandl et al, in an underestimation of the frequency of pDCs producing IFN-α in SMs. Indeed, in our hands addition of BFA after 2 or 4 hours of stimulation makes the detection of IFN-α-producing pDCs more sensitive in both SMs and RMs (data not shown). Interestingly, we were able to replicate the strong up-regulation of IFN-α mRNA levels that Mandl et al observed in similarly stimulated SM PBMCs (∼500-fold over baseline, see Mandl et al, Figure S3 [19]). Furthermore, we found that SM PBMCs show levels of IFN-β mRNA upregulation in response to TLR7 stimulation that are similar or even higher than those observed in RMs. The reasons why Mandl et al. attributed their initial finding of a “muted” IFN-α response at the protein level (which occurred in association with a robust mRNA upregulation) to purported IRF7 genetic defects, rather than post-transcriptional mechanisms, remain unclear. It is important to note that, in addition to the data presented here, in which we have shown robust production of IFN-α in pDCs in response to SIV directly, Harris and colleagues have also demonstrated that high levels of IFN-α production by pDCs in the LNs of SIV-infected SMs during acute infection are detectable using immunohistochemical techniques [21]. Taken together, these data indicate quite clearly that production of type I IFNs by SM pDCs is largely intact in SMs.

While the observed production of IFN-α by SM pDCs in response to SIV_mac239_ was quite robust, we found that it was significantly lower than in RMs in terms of frequency of pDCs producing IFN-α in response to SIV_mac239_. However, the relatively modest magnitude of this difference (41% vs 65%) makes it unlikely to play any major role in the divergent pathogenicity of SIV infection in these two species. This possibility becomes even more remote if one considers that the production of IFN-α by SM pDCs in response to SIV_mac239_, is in fact higher than that of human pDCs in response to HIV as observed by us (**Figure 2C**) and others [34], [45]. Of note, the difference in the frequency of pDCs producing IFN-α between SMs and RMs may be accounted for by the different kinetics of RNA production between the two species, with SMs resolving their IFN-α RNA upregulation more rapidly than RMs (**Figure 2**), and a consequently larger accumulation of intracellular IFN-α protein in RM pDCs during an overnight time course. In this regard, the current set of *in vitro* data is consistent with previously published *in vivo* data showing that acute SIV infection of SMs is associated with a massive, albeit transient, up-regulation of ISGs in both blood and lymphoid tissues [10], and confirms that a more effective down-modulation of type I IFN signaling, rather than an intrinsic genetic inability to produce this cytokine in response to TLR-7 ligands, is a candidate mechanism for the absence of chronic innate immune activation observed in SIV-infected SMs. This model is also consistent with the observation by several groups that acute SIV infection of another natural host species, the African green monkeys, is also associated with a robust but transient type I IFN response *in vivo* [46]–[48].

In our preliminary experiments, we also found that the ability of SM pDCs to produce IFN-α was highly sensitive to processing time, with a delay of even a few hours from collection to stimulation rendering these cells much less responsive to SIV (data not shown). This observation may in part explain the discrepancy between our results and those published by Mandl et al. In a subsequent report report [49], the same Authors describe a “muted” IFN-α response to yellow fever virus (YFV) both in vitro and in vivo. It will be important to establish if technical factors are also underlying these in vitro data, while the lower YFV replication in SMs as compared to RMs is the most parsimonious explanation for the lower in vivo IFN-α response to YFV observed in SMs in that experiment [49].

The data presented herein support a model in which the ability of SMs to avoid SIV-induced chronic immune activation is due, at least in part, to a rapid control of the type I IFN response occurring during acute infection. It is important to note, that a significant amount of work by several groups has demonstrated that SMs and AGMs have likely evolved multiple, non-mutually exclusive immunological strategies to avoid AIDS-defining chronic immune activation (reviewed in [3], [50]). While these mechanisms are only partly understood, they appear to be related to (i) an absence of chronic immune activation and (ii) preservation of key CD4+ lymphocyte subsets/function. The mechanisms underlying the maintenance of low immune activation of SIV-infected SMs are complex, and involve (i) the ability of SIVsmm Nef to effectively down-modulate the CD3-TCR complex from the surface of infected cells [51], [52]; (ii) the rapid down-modulation of the innate and adaptive immune activation associated with acute SIV infection [10], [21], coincident with a rapid up-regulation of PD-1 expression in LNs [53]; and (iii) the preservation of mucosal immune function, with normal levels of CD4+ Th17 cells in the intestine and absence of microbial translocation [54], [55]. In addition, we proposed that the observed lower levels of virus infection in CD4+ T_CM_ and in lymph nodes of SIV-infected SMs [31], [53] contributes to the low immune activation by compartmentalizing *in vivo* virus replication and antigen production away from the secondary lymphoid tissues in which most antiviral immune responses are initiated [3]. Similiarly, SIV_smm_ infected SMs also exhibit lower frequency of infection of lymph node resident CD4+ T_FH_ cells compared to SIV_mac239_ and SIV_E543_ infected RMs [53]. Preservation of CD4+ T helper function in natural hosts may also occur via CD4-negative ‘surrogates’. AGMs maintain a population of CD4-CD8α^DIM^ T cells demonstrating CD4+ T helper functionality [56], and SMs harbour a population of CD3+CD4-CD8- ‘double-negative’ T cells that exhibit a predominantly effector phenotype and share functional and transcriptomic features with T_H1_, T_H2_, T_H17_ and T_Fh_ [57]. This “double negative” lymphocyte population is preserved even in the rare SIV-infected SMs with extremely low (<50 cell/ul) CD4+ T cell counts [58] and may explain the lack of AIDS in these animals. While considerable progress has been made in our understanding of the virology and immunology of natural SIV infections, much more work is needed before a comprehensive and exhaustive model emerges of how SMs and other natural hosts avoid AIDS.

In conclusion, the data presented here demonstrate very clearly that SM pDC produce robust levels of type I IFNs in response to SIV, and that SM-IRF7 maintains intact IFNA transactivation and nuclear translocation activity. We believe that these findings resolve a previous significant theoretical contradiction in the field of natural SIV infection and provide a solid premise for future studies aimed at defining the molecular mechanisms by which innate immune responses to SIV are rapidly down-modulated in natural host species despite ongoing virus replication. Ultimately, it is hoped that these advances may help designing interventions that target the chronic innate immune activation that is associated with HIV infection of humans.

## Materials and Methods

### Ethics statement

Ten healthy HIV-uninfected individuals were recruited for this study for blood draws. All individuals who participated in this study provided informed consent in writing in accordance to the protocol approved by the Institutional Review Board of Emory University, IRB#00045821, entitled “Phlebotomy of healthy adults for the purpose of evaluation and validation of immune response assays”. The protocol adheres to international guidelines established in the Declaration of Helsinki by the World Medical Association. Blood draws were obtained from sooty mangabeys and rhesus macaques housed at the Yerkes National Primate Research Center, which is accredited by American Association of Accreditation of Laboratory Animal Care. This study was performed in strict accordance with the recommendations in the Guide for the Care and Use of Laboratory Animals of the National Institutes of Health, a national set of guidelines in the U.S. and also to international recommendations detailed in the Weatherall Report (2006). This work received prior approval by the Institutional Animal Care and Use Committees (IACUC) of Emory University (IACUC protocol #2000793, entitled “Comparative AIDS Program”). Appropriate procedures were performed to ensure that potential distress, pain, discomfort and/or injury was limited to that unavoidable in the conduct of the research plan. The sedative Ketamine (10 mg/kg) and/or Telazol (4 mg/kg) were applied as necessary for blood draws and analgesics were used when determined appropriate by veterinary medical staff.

### IRF7 sequencing

16 sooty mangabeys (*Cercocebus atys atys*) were identified from different families housed at the Yerkes National Primate Research Center (YNPRC) that cumulatively have over 50 offspring within the group and thus represent a large proportion of the genetic variance within the sooty population. Added to this group was animal FHy, from which the IRF7 sequence in the Mandl et al. [19] study was originally derived. Exonic sequence of the IRF7 gene was obtained from UCSC genome browser (http://genome.ucsc.edu/cgi-bin/hgGateway) using the rheMac2 assembly of the Indian rhesus macaque genome, and primers were designed to span the entire exon by Primer3 (http://frodo.wi.mit.edu). PCR was performed using standard amplification reactions on ABI 9700 thermal cyclers using MgCl2 concentrations of either 1.5 mM or 2.0 mM. PCR products were checked for expected size by electrophoresis on agarose gels. Shrimp alkaline phosphatase and Exonuclease I were added to remove single stand DNA. Direct Sanger sequencing reactions were performed using Applied Biosystem Big Dye terminator protocol on an ABI 9700 thermal cycler. The reaction was purified by EDTA/EtOH protocol, and sequencing reactions performed on an ABI 3730 genetic analyzer. Subsequent analysis was done using Sequencher 4.7 genetic software. Results were aligned to the corresponding rhesus genomic location. Sequence products of all exons mapped to the expected genomic locations. In secondary analysis we sequenced all 177 animals that currently make up the Yerkes NPRC sooty mangabey colony for the exons exhibiting variation at the amino acid level using the same techniques (**Smentary Table S1**). All reported sequences were submitted to NCBI GenBank (**Supplementary Table S2**). One-way ANOVA was used to compare the effect of each SNP genotype upon the listed phenotypic measures of immune function.

### Plasmids

N-terminal FLAG fusion proteins of human-IRF7 and the 6D, 7A, Δ12-101 mutants, TBK1 and the IFNA4-luciferase reporter have been described previously [26], [27]. The full-length coding region of sooty mangabey IRF7 containing the most representative alleles (animal FFz) within the colony was synthesized by Invitrogen(Carlsbad, CA), rhesus IRF7 was PCR amplified from cDNA; and both were cloned into the pFLAG-CMV2 vector using the HindIII and BamHI sites with n-terminal FLAG sequences. The nt sequence of FFz IRF7 has been posted to GenBank under accession # JX438328; the nucleotide, amino acid translation, and alignment of FFz smIRF7 aligned with rhesus and human IRF7 is available in the **Supplementary Data Files S1, S2, S3**. The pGL3 reporter plasmid (SV40-luciferase) was purchased from Promega (Madison, WI). eGFP-SM-IRF7 was made by subcloning sooty mangabey IRF7 into a parental vector of pEGFP-C1 Hu-IRF7 described previously [28]. Subcloning of IRF7 was performed by the Emory Custom Cloning Core Facility.

### Transfections, luciferase and GFP assays

HEK-293 cells were maintained in Dulbecco's modified Eagle's medium supplemented with 10% fetal bovine serum (FBS). For transient transfection, the HEK-293 cells were cotransfected in 24-well plates with 50 ng of IFNA promoter reporter plasmid, 50 ng of TBK1 and 50 ng of various IRF7 plasmids. All transfections were done with Lipofectamine 2000 (Invitrogen) as detailed by the manufacturer using 150 ng of plasmid in 70 ul transfection cocktails. The pGL3 plasmid served as positive control and empty vector was used to equalize the total amount of DNA. Luciferase assays were performed with the Luciferase Assay System (Promega). Cells were harvested 24 hrs posttransfection and lysed in passive lysis buffer and frozen at −80 C. 20 ul of lysates were mixed with Promega Luciferase Assay Substrate and read by luminometer. Readings for a media control were subtracted from relative light unit readings for individual samples. For IRF7-GFP visualization studies, COS cells were maintained in DMEM with 10% FBS and plated to approximately 80% confluency in 6-well plates. 4 ug total plasmid was transfected using 500 ul of 2% Lipofectamine in RPMI. After 24 hrs, cells were washed with PBS, fixed with ice cold 1∶1 methanol∶acetone at −80 C for 10 min and reconstituted in PBS with ProLong Gold Antifade Reagent with DAPI and transferred to slides. Localization was visualized on an AxioVision image capture system (Carl Zeiss) fluorescent microscope at 200× and 630× magnification.

### Virus and TLR stimulations

PBMCs were obtained via density gradient centrifugation using Lymphocyte Separating Media (Lonza) according to manufacturer's instructions. Immediately following isolation, cells were cultured in a 48-well plate at 4×10^6^ cells/ml in media containing RPMI (CellGro), 10% defined fetal bovine serum (HyClone) penicillin-streptomycin (CellGro), and L-glutamine (CellGro), and incubated with 1 ng/ml IL-3 (R&D Systems) for 30 min at 37°C and 5% CO_2_. Cells were then stimulated with 10 µM CL097 (Invivogen) or virus, as described. All viral stimulations (SIVmac239, SIVsme543.1 or HIV-1 ADA) were conducted using aldrithiol 2 (AT-2) inactivated strains at a concentration of 3 µg/ml (provided by Dr Jeff Lifson, NCI, NIH) overnight at 37°C and 5% CO_2_. For intracellular cytokine experiments, Brefeldin-A (10 µg/ml) was added after 2 hrs of stimulation unless otherwise indicated.

### Flow cytometry and intracellular cytokine staining

Intracellular cytokine staining was performed following the protocol described by Lamoreaux et al [59]. Briefly, PBMCs were incubated with Aqua Live/Dead amine dye-AmCyan (Invitrogen) for 10 min followed by 30 min incubation with antibodies specific for surface markers. For cytokine detection PBMCs were fixed and permeabilized using Cytofix/Cytoperm reagents (BD Biosciences) according to manufacturer's instructions. Cells were stained with monoclonal antibodies to the following proteins: anti-CD123 PE-Cy7 (clone 6H6) (eBioscience), anti-CD20 Pacific Blue (clone 2H7) (BioLegend), anti-CD14 PE-Texas Red (clone RM052) (Beckman Coulter), anti-IFNa-2 PE (clone 225.c) (Chromaprobe), Aqua LiveDead (Invitrogen), and anti-CD3 Pacific Blue (clone SP34-2), anti-CD11c APC (clone SHCL-3), anti-HLA-DR PerCP-Cy5.5 (clone L243/G46-6) (all from BD Biosciences). At least 1,500,000 single, live lymphocyte events were acquired on a LSRII flow cytometer (BD). Analysis was performed with FlowJo (Treestar). pDCs were classified through the following gates: singlet, lymphocyte, CD3-CD20-HLADR+CD14-CD11c-CD123+ as shown in **Supplementary Figure S1**.

### RNA purification

Total RNA from PBMCs was purified using RNeasy mini kits (QIAGEN) according to manufacturer's protocol utilizing on-column DNAse digestion. RNA quantity was estimated using Nanodrop analysis.

### Real time PCR

RNA samples (0.5–1.0 µg) were reverse transcribed in a volume of 20 µl as previously described [9] and 0.1 µl of cDNA was used for real time PCR analysis using an ABI 7900 HT instrument (Applied Biosystems). Primers specific for GAPDH mRNA were used to normalize samples. Fold-changes were calculated using the relative standard curve method. IFN-α mRNA was measured using TAQman probes from Applied Biosystems and Universal MasterMix and IFN-β was quantitated using SYBR green. ABI TAQman Probes were: rhesus IFNa2 (Rh029027494); rhesus GAPDH (Rh02621745); Primer sequences for SYBR green PCR were: IFNB-forward 5′-TTC GCT CTG GCA CAA CAG GTA GTA -3′, IFNB-reverse 5′-AGC CTT CCA TTC AAT TGC CAC AGG-3′; GAPDH-forward 5′-GAA GGT GAA GGT CGG AGT C, GAPDH-forward 3′-CAA GCT TCC CGT TCT CAG CC.

### IFN-β ELISA

PBMCs from RMs and SMs were incubated with 10 µM CL097 for the times indicated, after which 100 µl of supernatant was harvested. IFN-β levels were quantitated using the Simian Interferon Beta kit (USCN) according to manufacturer's instructions. Samples were incubated on pre-coated plates in duplicate. Plates were then read at 405 nm within 15′ on a VMax Kinetic microplate reader (Molecular Devices). A standard was used to quantity protein levels and blanks were used to determine background absorbance. MPMIII (Biorad) was used to analyze data.

## Supporting Information

Data File S1 **Alignment of smIRF7 representative animal (FFz) with human and rhesus.**
(RTF)

Data File S2 **Amino acid sequence of smIRF7 from consensus animal FFz in FASTA file format.**
(RTF)

Data File S3 **Nucleotide sequence of smIRF7 coding region from consensus animal FFz in FASTA file format.**
(RTF)

Figure S1 **Gating strategy for pDCs and intracellular IFN-α production.** PBMCs from SMs, RMs, and humans were incubated 18 hrs with SIV, HIV or CL097, and then stained for intracellular IFN-α. The gating strategy to enumerate pDCs is shown; after exclusion of doublets and large, granular cells, pDCs were identified as CD3,CD20,CD14,CD11c-negative, and HLA-DR, CD123 positive. CD123 hi, CD11c- cells were gated and enumerated for IFN-α production. >98% of cells producing IFN-α were gated within the pDC population (data not shown).
(EPS)

Figure S2 **IFN-α production in reponse to SIVsmE543.1.** Percentage of IFN-α+ pDCs after 18 hr incubation of 3 ug/ml AT2 SIV_smE543.1_ with PBMCs from RMs and SMs. Means are shown by horizontal bars.
(EPS)

Table S1 **Number of sooty mangabeys sequenced for each of the IRF7 exons.**
(DOC)

Table S2 **GenBank Accession Numbers for IRF7 coding region nucleotide sequences from Sooty Mangabeys in the colony at the Yerkes National Primate Research Center.**
(XLS)
