# Supplementary material for: Intact Type I Interferon Production and IRF7 Function in Sooty Mangabeys
Source: PLoS Pathog. 2013 Aug 29;9(8):e1003597. doi: 10.1371/journal.ppat.1003597 (PMC3757038; doi:10.1371/journal.ppat.1003597)
Supplement: Data File S1 — Alignment of smIRF7 representative animal (FFz) with human and rhesus. (RTF) [file ppat.1003597.s001.rtf]

Supplementary Data S1 - Alignment of smIRF7 representative animal (FFz) with human and rhesus.Homo_sapiens_IRF7_isoform_a_refseq      MALAPERAAPRVLFGEWLLGEISSGCYEGLQWLDEARTCFRVPWKHFARKDLSEADARIFMacaca_Mulatta_IRF7_refseq              MALAPERAAPRVLFGEWLLGEISSGCYEGLQWLDEARTCFRVPWKHFARKDLSEADARIFFFz_smIRF7                              MALAPERAAPRVLFGEWLLGEISSGCYEGLQWLDEARTCFRVPWKHFARKDLSEADARIF                                        ************************************************************Homo_sapiens_IRF7_isoform_a_refseq      KAWAVARGRWPPSSRGGGPP-PEAETAERAGWKTNFRCALRSTRRFVMLRDNSGDPADPHMacaca_Mulatta_IRF7_refseq              KAWAVARGRWPPSSRGGDPPPPEAEAAERAGWKTNFRCALRSTRRFVMLRDNSGDPADPHFFz_smIRF7                              KAWAVARGRWPPSSRGGDPPPPEAEAAERAGWKTNFRCALRSTRRFVMLRDNSGDPADPH                                        ***************** ** ****:**********************************Homo_sapiens_IRF7_isoform_a_refseq      KVYALSRELCWREGPGTDQTEAEAPAAVPPPQGGPPGPFLAHTHAGLQAPGPLPAPAGDKMacaca_Mulatta_IRF7_refseq              KVYALSPELGWREGPGTDQTEAEGPAAVRPPQGRPPGPFLAHRDAGLQAPGPFPAPAGDKFFz_smIRF7                              KVYALSPELGWREGPGTDQTEAEAPAAVRPPQGRPPGPFLAHRDGGLQAPGPLPAPAGDK                                        ****** ** *************.**** **** ******** ..*******:*******Homo_sapiens_IRF7_isoform_a_refseq      GDLLLQAVQQSCLADHLLTASWGADPVPTKAPGEGQEGLPLTGACAGGPGLPAGELYGWAMacaca_Mulatta_IRF7_refseq              GDLLLQAVQQSCLADHLLTASWGADPVPAQAPGEGQEGLPLTGACAGGPGLPAGELCTWAFFz_smIRF7                              GDLLLQAVQQSCLADHLLTASWAADPVPAQAPGEGQEGLPLTGACAGGPGLPAGELCTWA                                        **********************.*****::**************************  **Homo_sapiens_IRF7_isoform_a_refseq      VETTPSPGPQPAALTTGEAAAPESPHQAEPYLSPSPSACTAVQEPSPGALDVTIMYKGRTMacaca_Mulatta_IRF7_refseq              VEATPSPGPQPTALMTGEATAPEPPHQAEPYLAPSPSACTVVQEPSPGALDVTIMYKGRTFFz_smIRF7                              VEATPSPGPQPAALMTGEATAPEPPHQVEPYLAPSPSACTAVQEPSPGALDVTIMYKGRT                                        **:********:** ****:*** ***.****:*******.*******************Homo_sapiens_IRF7_isoform_a_refseq      VLQKVVGHPSCTFLYGPPDPAVRATDPQQVAFPSPAELPDQKQLRYTEELLRHVAPGLHLMacaca_Mulatta_IRF7_refseq              VLQKVVGHPSCMFLYGPPDPAVRATDPQQVAFPSPAELPDQKQLRYTEELLRHVAPGLQLFFz_smIRF7                              VLQKVVGHPSCMFLYGPPDPAVRATDPQQVAFPSPAELPDQKQLRYTEELLRHVAPGLQL                                        *********** **********************************************:*Homo_sapiens_IRF7_isoform_a_refseq      ELRGPQLWARRMGKCKVYWEVGGPPGSASPSTPACLLPRNCDTPIFDFRVFFQELVEFRAMacaca_Mulatta_IRF7_refseq              ELRGPQLWARRMGKCKVYWEVGGPPGSASPSTPACLLPRNCDTPIFDFRVFFQELVEFRAFFz_smIRF7                              ELRGPQLWARRMGKCKVYWEVGGPPGSASPSTPACLLPRNCDTPIFDFRVFFRELVEFRA                                        ****************************************************:*******Homo_sapiens_IRF7_isoform_a_refseq      RQRRGSPRYTIYLGFGQDLSAGRPKEKSLVLVKLEPWLCRVHLEGTQREGVSSLDSSSLSMacaca_Mulatta_IRF7_refseq              RQRRGSPCYTIYLGFGQDLSARRPKEKSLVLVKLEPWLCRVHLEGTQREGVSSLDSSSLSFFz_smIRF7                              RQRRGSPCYTIYLGFGQDLSARRPKEKSLVLVKLEPWLCRVHLEGTQREGVSSLDSSSLS                                        ******* ************* **************************************Homo_sapiens_IRF7_isoform_a_refseq      LCLSSANSLYDDIECFLMELEQPAMacaca_Mulatta_IRF7_refseq              LCLSSTNSLYDDIECLLMELEQPVFFz_smIRF7                              LCLSSTNSLYDDIECLLMELEQPV                                        *****:*********:*******.
