# Supplementary material for: Intact Type I Interferon Production and IRF7 Function in Sooty Mangabeys
Source: PLoS Pathog. 2013 Aug 29;9(8):e1003597. doi: 10.1371/journal.ppat.1003597 (PMC3757038; doi:10.1371/journal.ppat.1003597)
Supplement: Data File S2 — Amino acid sequence of smIRF7 from consensus animal FFz in FASTA file format. (RTF) [file ppat.1003597.s002.rtf]

>smIRF7_consensus_FFz_aaMALAPERAAPRVLFGEWLLGEISSGCYEGLQWLDEARTCFRVPWKHFARKDLSEADARIF KAWAVARGRWPPSSRGGDPPPPEAEAAERAGWKTNFRCALRSTRRFVMLRDNSGDPADPH KVYALSPELGWREGPGTDQTEAEAPAAVRPPQGRPPGPFLAHRDGGLQAPGPLPAPAGDK GDLLLQAVQQSCLADHLLTASWAADPVPAQAPGEGQEGLPLTGACAGGPGLPAGELCTWA VEATPSPGPQPAALMTGEATAPEPPHQVEPYLAPSPSACTAVQEPSPGALDVTIMYKGRT VLQKVVGHPSCMFLYGPPDPAVRATDPQQVAFPSPAELPDQKQLRYTEELLRHVAPGLQL ELRGPQLWARRMGKCKVYWEVGGPPGSASPSTPACLLPRNCDTPIFDFRVFFRELVEFRA RQRRGSPCYTIYLGFGQDLSARRPKEKSLVLVKLEPWLCRVHLEGTQREGVSSLDSSSLS LCLSSTNSLYDDIECLLMELEQPV
