# Supplementary material for: Intact Type I Interferon Production and IRF7 Function in Sooty Mangabeys
Source: PLoS Pathog. 2013 Aug 29;9(8):e1003597. doi: 10.1371/journal.ppat.1003597 (PMC3757038; doi:10.1371/journal.ppat.1003597)
Supplement: Data File S3 — Nucleotide sequence of smIRF7 coding region from consensus animal FFz in FASTA file format. (RTF) [file ppat.1003597.s003.rtf]

>smIRF7_consensus_FFz_nt_GENBANK:JX438328ATGGCCTTGG CTCCTGAGAG GGCGGCCCCA CGCGTGCTGT TCGGAGAGTG GCTCCTTGGA GAGATCAGCA  GCGGCTGCTA TGAGGGGCTG CAGTGGCTGG ACGAGGCCCG CACCTGCTTC CGCGTGCCCT GGAAGCACTT CGCGCGCAAG GACCTGAGCG AGGCTGACGC GCGCATCTTC AAGGCCTGGG CCGTGGCCCG CGGCAGGTGG CCGCCTAGCA GCAGAGGAGG TGACCCGCCG CCCCCCGAGG CTGAGGCTGC GGAGCGCGCC GGCTGGAAAA CCAACTTCCG CTGCGCACTG CGCAGCACGC GCCGCTTCGT GATGCTGCGA GATAACTCGG GGGACCCGGC CGACCCGCAC AAGGTGTACG CGCTGAGCCC GGAGCTGGGC TGGCGAGAAG GCCCAGGCAC GGACCAGACT GAGGCAGAGG CCCCCGCGGC TGTCCGGCCA CCGCAGGGCA GGCCCCCAGG GCCATTCCTG GCACACAGAG ATGGTGGACT CCAAGCCCCA GGCCCCCTCC CTGCCCCAGC TGGTGACAAG GGGGACCTCC TGCTCCAGGC AGTGCAACAG AGCTGCCTGG CGGACCATCT GCTGACAGCG TCATGGGCGG CAGACCCAGT CCCAGCCCAG GCTCCTGGAG AGGGACAAGA GGGTCTTCCC CTGACTGGGG CCTGTGCTGG AGGTCCAGGG CTCCCTGCTG GGGAGCTGTG CACATGGGCA GTAGAAGCAA CCCCTAGCCC CGGGCCCCAG CCCGCGGCGC TAATGACAGG CGAGGCCACG GCCCCAGAGC CCCCGCACCA GGTAGAGCCA TACCTGGCAC CCTCCCCAAG TGCCTGCACT GCGGTGCAAG AGCCCAGCCC AGGGGCGCTG GACGTGACCA TCATGTACAA GGGCCGCACA GTGCTACAGA AGGTGGTGGG GCACCCGAGC TGCATGTTCC TGTACGGCCC CCCAGACCCA GCTGTCCGGG CCACAGACCC CCAGCAGGTA GCATTCCCCA GCCCTGCTGA GCTCCCCGAC CAGAAGCAGC TGCGCTACAC GGAGGAACTG  CTGCGGCATG TGGCCCCTGG GCTGCAGCTG GAGCTTCGGG GGCCACAGCT GTGGGCCCGG CGCATGGGCA AGTGCAAGGT GTACTGGGAG GTGGGTGGCC CCCCGGGCTC CGCCAGCCCC TCCACCCCAG CCTGCCTGCT GCCTCGGAAC TGCGACACCC CCATCTTTGA CTTCAGAGTC TTCTTCCGAG AGCTGGTGGA ATTCCGGGCA CGGCAGCGCC GCGGCTCCCC CTGCTATACC ATCTACCTGG GCTTCGGGCA GGACCTGTCA GCCAGGAGGC CCAAGGAGAA GAGCCTGGTC CTGGTGAAGC TGGAGCCCTG GCTGTGCCGA GTGCACCTGG AGGGCACGCA GCGTGAGGGT GTGTCTTCCC TGGATAGTAG CAGCCTCAGC CTCTGTCTGT CCAGCACCAA CAGCCTCTAT GATGACATTG AGTGTTTGCT CATGGAGCTG GAGCAGCCC  GTCTAG
