# Supplementary material for: Intact Type I Interferon Production and IRF7 Function in Sooty Mangabeys
Source: PLoS Pathog. 2013 Aug 29;9(8):e1003597. doi: 10.1371/journal.ppat.1003597 (PMC3757038; doi:10.1371/journal.ppat.1003597)
Supplement: Table S1 — Number of sooty mangabeys sequenced for each of the IRF7 exons. (DOC) [file ppat.1003597.s006.doc]

**Supplementary Table 1**. Number of sooty mangabeys sequenced for each of the IRF7 exons.

| IRF 7 Exon | Rhesus Genomic location | Animals Sequenced |
| --- | --- | --- |
| 1 | chr14:423655-423674 | 16 breeders |
| 2 | chr14:423407-423569 | 177 animals |
| 3 | chr14:423110-423323 | 177 animals |
| 4 | chr14:422499-422557 | 16 breeders |
| 5 | chr14:422197-422422 | 177 animals |
| 6 | chr14:421962-422048 | 50 animals |
| 7 | chr14:421796-421876 | 177 animals |
| 8 | chr14:421334-421723 | 177 animals |
| 9 | chr14:421127-421245 | 16 breeders |
| 10 | chr14:420886-421041 | 16 breeders |
